# Just Research: Evaluation findings of an educational program to promote inclusive research among investigators and research staff

**DOI:** 10.1017/cts.2024.592

**Published:** 2024-09-16

**Authors:** Susan Racine Passmore, Gina Green-Harris, Peter Kirschmann, Amarette Filut, Katrina Phelps, Mariana Garcia, Dorothy Farrar Edwards

**Affiliations:** 1 School of Nursing, University of Wisconsin, Madison, WI, USA; 2 Collaborative Center for Health Equity, Institute for Clinical and Translational Research, School of Medicine and Public Health, University of Wisconsin, Madison, WI, USA; 3 Center for Community Engagement and Health Partnerships, School of Medicine and Public Health, University of Wisconsin, Madison, WI, USA; 4 Department of Medicine, School of Medicine and Public Health, University of Wisconsin, Madison, WI, USA; 5 Professional Learning and Community Education (PLACE) University of Wisconsin, Madison, WI, USA; 6 Wisconsin Network for Research Support, School of Nursing, University of Wisconsin, Madison, WI, USA; 7 Department of Kinesiology, School of Education, University of Wisconsin, Madison, WI, USA

**Keywords:** Recruitment, health equity, inclusion, diversity, researcher education

## Abstract

Educational opportunities for investigators and staff to promote inclusive research practices are a critical piece of the effort to increase diversity in study participation and promote health equity. However, few trainings to date have empirically been shown to result in behavior changes. We present preliminary evaluation findings for the *Just Research* workshop offered at the University of Wisconsin–Madison between October 2022 and August 2023. These sessions included 80 participants who made up 4 cohorts. Data was collected through a retrospective pre/post-test survey administered 0–7 days following the workshop (*n* = 70), and a follow-up survey administered 9–12 months following the workshop (*n* = 21). Participants demonstrate significant increases in knowledge and self-efficacy regarding implementing inclusive practices post-intervention (*p* < .001). 85.7% of participants who completed the follow-up survey reported implementing inclusive practices.

## Introduction

The ongoing lack of diversity in research participation limits our ability to progress toward health equity, as the very communities experiencing health disparities are those underrepresented in research [1,2]. While there have been many explorations of the problem, most have focused on a lack of trust in research on the part of communities experiencing health disparities, focusing on the problem outside of the research community [3]. However, recent attention has been paid to the barriers to diversity in research participation borne of researcher behaviors and practices. For example, recent publications from Wilkins, Manly, et al. and Gilmore-Bykovskyi et al. seek to understand, problematize, and, ultimately, reduce the use of “exclusionary research practices” in research [4–6]. Such practices can include but are not limited to lack of attention to the needs of potential participants from a range of backgrounds, withholding opportunities to participate in research from individuals who are not perceived as “good” participants, using needlessly narrow eligibility requirements that affect groups disproportionately (e.g., lack of comorbidities) or setting all recruitment/enrollment efforts in places where there is an existing lack of diversity such as academic health centers [2,6,7]. Our own mixed-methods exploration of attitudes of clinical investigators revealed a high level of recognition of the problem but a low level of actual implementation of inclusive practices [8,9]. Interviews revealed ideas about who makes a “good” research participant as well as the perception of the problem as unsolvable. These findings, as do those of Niranjan et al. and others, indicate a need for researcher education [10–12].

With the identification of researcher barriers, several educational programs have been developed to build capacity for inclusive research engagement. An early example of this was the *Building Trust between Minorities and Researchers* program (2012–2021), which consisted of a two-day workshop focused on raising awareness of research abuse and participant perspectives of mistrust [13]. Another recent effort is a massive open online course called *Faster Together, Enhancing the Recruitment of Marginalized Communities in Clinical Trials* [14]. Faster Together is designed for a general audience involved in health-related research recruitment in clinical or community settings. Eight modules featuring videos, text, and quizzes are offered for an audience to move through at their own pace. A study evaluating the Faster Together course demonstrated increased knowledge and intention to change research practices based on pre- and post-test data collected in the first 10 months since the course was released. While 382 individuals enrolled in the course during the first ten months, only 105 participants completed the pre-test, and 14 completed the post-test. Another program is a certification program for clinical research coordinators that includes a series of 1–2 hour sessions, including *Just Ask: Equity and Diversity in Clinical Research,* as well as more general recruitment educational sessions, such as *Using Social Marketing Principles to Design Your Engagement Strategy* [15]. Evaluation of the Duke certificate program has revealed impressive outcomes, including self-reported competency and manager-reported skill level increases.

In the article, we report on preliminary outcomes from yet another program, the *Just Research* workshop, established at the end of 2022. Like the programs discussed above, *Just Research* seeks to promote the capacity for the engagement of participants from groups underrepresented in research. Data collection has included a retrospective pre/post-test and follow-up surveys.

## Methods and materials

### Just Research workshop

This workshop was developed at the University of Wisconsin–Madison based on our assessment of barriers to inclusive practices on our campus among investigators [8] and staff and our experience as members of the *Building Trust* team [13]. *Just Research* promotes holistic, intentional approaches that prioritize inclusivity by focusing on (1) bias recognition and reduction, (2) the incorporation of community perspectives in research design and procedures, (3) engagement best practices, and (4) critical practice with translating new skills into potential changes in practice relative to each participant’s work [8,9]. Workshop learning objectives are to understand opportunities within the academic research community to promote diversity in research participation and engagement; to identify principles that guide responsible, respectful, and sustainable engagement; to explore avenues to increase community voice in research participation based on engagement and recruitment science; and to develop practical ways to integrate new skills into one’s work. Through interactive exercises based on real-world experiences and opportunities to explore ways to tailor best practices to their individual needs, *Just Research* participants can explore researcher “trustworthiness” and build practical strategies to promote inclusivity [4,6,16,17]. The program is available for investigators and research staff (teams are encouraged to participate together). Workshops are full-day, face-to-face, and kept small (∼18–22 participants) to facilitate discussion and activities. To date, we have provided four workshops (four cohorts) in October 2022, February, June, and August of 2023. All have been jointly facilitated by the program’s co-directors (GG-H and SRP), who have considerable experience in academic/community communication and partnership. All participants included in the evaluation reported here received the same curriculum in the same format. To enroll in the workshop, participants self-selected and responded to an open registration link distributed across the University of Wisconsin (UW) Institute for Clinical and Translational Research (ICTR) affiliates and promoted on the ICTR website and social media platforms. Some participants signed up as a team; others registered as individuals.

### Just Research evaluation

**Figure 1.**
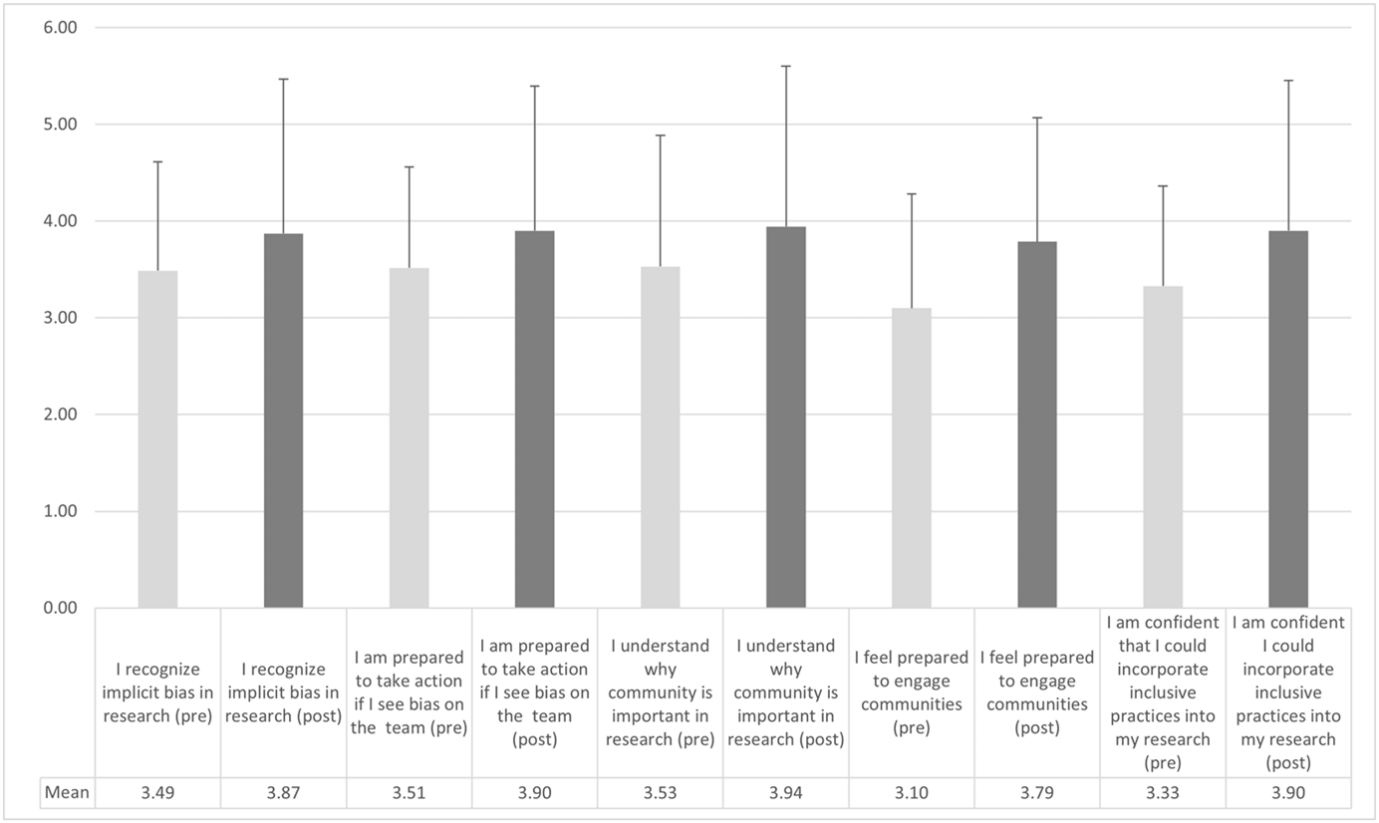
Participant mean scores on individual items – pre- and post-tests. (*n* = 70).

As our previous work had indicated the lack of self-efficacy regarding inclusive research practices as a barrier [8], the *Just Research* evaluation is specifically designed to address this outcome along with knowledge and skills. We collect both process and outcome-level data, including information on participants’ roles in research, years of research, and experience in clinical research. Surveys are optional and were distributed immediately following the workshop. Participants received three reminders over the following weeks. We tracked outcomes through a retrospective pre-post survey to assess participants’ self-reported attitudes, skills, and self-efficacy on Likert-scale items after the session. Retrospective pre-tests are helpful to avoid a response-shift basis, which can occur when participants’ frames regarding a topic so shift from the experience of the training itself that traditional pre-test/post-test design can essentially measure different phenomena (e.g., participants overestimate knowledge and skills on traditional pre-test and underestimate at post-test after realizing that the topic more complex than previously believed) [18–20]. Main outcomes for pre-post comparison centered around specific inclusive behaviors, including the recognition of bias on the research team, taking action to correct the bias, understanding the importance of community perspective, seeking out community perspectives through partnerships or other relationships, and general ability to integrate inclusive practices (e.g., maximize recruitment through a range of venues), which reflect curriculum objectives. Ongoing change and adoption of inclusive behaviors (e.g., implementation of community-engaged strategies, review of exclusion criteria, and recruitment plans to maximize diverse participation) were assessed at the 9–12-month follow-up with a brief survey also using Likert-scale items and some open-ended questions. All surveys were pilot tested with 8–10 investigators and research staff to ensure that participants clearly and correctly interpreted items. This could only be assessed from cohorts 1 and 2, who participated in the workshop in October 2022 and February 2023 (other cohorts will complete follow-up surveys later in 2024). We created a composite scale of self-assessed knowledge, skills, and self-efficacy from five items (see Figure 1) in the pre- and post-tests in IBM SSPS to allow for the comparison. Reliability was assessed using Cronbach’s alpha coefficient (α 0.85 and 0.98, respectively). Scale scores were compared using a two-tailed *t*-test. Due to the limited number of follow-up surveys to date, we only present descriptive statistics. All study activities have been reviewed and approved by the University of Wisconsin IRB (ID: 2024-0053)

## Results

In all, 78 investigators and staff participated in 4 workshops. Of these, 70 provided pre/post-data, and 21 participated in follow-up surveys (distributed 9–12 months post-workshop). Table 1 illustrates the characteristics of our 70 workshop participants who have participated in the pre/post-test to date. The majority are affiliated with the School of Medicine and Public Health and identified themselves as research staff. However, we did have participation from investigators as well (23%).

**Table 1.** Survey participant characteristics (*n* = 70)

Item		Pre/post (*n* = 70)	Follow-up (*n* = 21)
Primary institution	UW–Madison	97.1% (68)	95.3% (20)
	UW Health	1.4% (1)	4.7% (1)
	Other	1.4% (1)	–
If UW–Madison, School or College	School of Medicine and Public Health	79.4% (54)	55% (11)
	School of Nursing	11.8% (8)	30% (6)
	School of Pharmacy	5.9% (4)	15% (3)
	Other	2.9% (2)	–
How long have you been involved in human subject research in some capacity?	Less than one year	2.9% (2)	–
	1–5 years	27.1% (19)	–
	6–10 years	37.1% (26)	52.4% (11)
	11–20 years	18.6% (13)	23.8% (5)
	More than 20 years	14.3% (10)	23.8% (5)
Roles in research studies involving human subjects? (check all that apply)	Investigator (PI or Co-I)	22.9% (16)	42.8% (9)
	Clinical trials coordinator	32.9% (23)	23.8% (5)
	Project manager	20.0% (14)	–
	Recruitment/outreach staff	32.9% (23)	28.6% (6)
	Other study staff	38.6% (27)	38.1% (8)
Gender	Man	10.0% (7)	4.7% (1)
	Woman	88.6% (62)	90.6% (19)
	Nonbinary	1.4% (1)	4.7% (1)
Identify as Hispanic or Latino	Yes	8.6% (6)	4.7% (1)
Which of the following describes how you self-identify regarding race?	American Indian or Alaskan Native	–	–
	Asian	11.4% (8)	4.7% (1)
	Black or African American	18.6% (13)	23.8% (5)
	Native Hawaiian or other Pacific Islander	–	–
	White	70.0% (49)	47.6% (14)
	Other	4.3% (3)	4.7% (1)

Participants were asked five key questions designed to assess their level of self-assessed knowledge, skills, and self-efficacy. Across all these items, we found improvement in the comparison of means pre- and post-test (see Figure 1).

In comparing retrospective pre/post-test knowledge, skill, and self-efficacy scales using paired sample *t*-tests, we find a modest but statistically significant difference across all items (*t* −4.266, SD 0.96504, *p* < .001). A relatively high standard deviation for each item indicates the wide range of participant skills, knowledge, and self-efficacy both before and after the workshop.

Post-tests included open-ended items requesting workshop feedback, which was largely positive. For example,

*It was a wonderful experience, and I will be recommending it to the rest of my research team.*

And,

*It should be required for people doing human subjects research.*

The 9–12-month follow-up survey was completed by 21 of the 38 (55% response rate) participants in cohorts 1 and 2, as they were the only cohorts who completed the program in the follow-up timeframe. There is no reason to believe that these cohorts are unique in comparison to the following cohorts. Data on self-reported inclusive research behaviors tied to their participation in a *Just Research* workshop is presented in Table 2.

**Table 2.** Follow-up survey response at 9–12 months post-session (*n* = 21)

	Strongly/ somewhat disagree	Neither agree nor disagree	Somewhat/ strongly agree
I’ve thought about the content of the Just Research workshop several times since I participated.	4.8% (1)	–	95.2% (20)
I’ve talked to colleagues about the Just Research content or the workshop since I participated.	14.3% (3)	9.5% (2)	76.2% (16)
I have been able to recognize different forms of implicit bias in my research practice.	4.5% (1)	9.1% (2)	85.7% (18)
I have taken action when I perceived bias or racism on the research team or in interactions with participants.	–	23.8% (5)	76.2% (16)
I think about community perspectives when considering a research project.	–	–	100% (21)
I have taken steps to engage communities in research, including those who have been historically underrepresented.	4.8% (1)	23.8% (5)	71.4% (15)
I have worked to incorporate more inclusive practices into my research.	4.8% (1)	9.5% (2)	85.7% (18)

76.2% of participants in the follow-up survey attributed changes in their work as a direct result of their participation in *Just Research*. In response to an open-ended question about the specifics of such changes, participants reported increased awareness of bias for themselves and, notably, their teams. For some, participation led to more open communication regarding inclusion on the research team. For example,

*From my position as a Research Program Assistant, I find myself more comfortable with questioning PI practices and making suggestions for how to open the work to community partners.*

Some participants also reported the establishment of new partnerships or community advisory boards. For example,

*I have begun making meaningful and bidirectional relationships with members and groups associated with my field of study. Instead of engaging them when I need something, I shifted my focus to curating genuine friendships and relationships.*

And,

*[We have] more intentional engagement and collaboration to serve historically excluded and underrepresented communities.*

Others reported changes in recruitment or other research practices. For example,

*I’m now offering recruitment flyers at community locations outside healthcare clinics and have spoken with stakeholders about ways to improve my recruitment practices.*

And,

*Thoughtfully read through consent to try to ensure that participants are informed. Made modifications to consent and subject facing material if it was not clear or misleading.*

## Discussion

The path to increasing diversity in research participation is multifaceted and will necessarily include shifts in the attitudes and behaviors of members of our research community. Intentionally or not, there are many ways we, as researchers, “get in our own way” [8,10,21]. For example, driven by traditional approaches that govern eligibility criteria, recruitment sites, and ideas about who is willing and able to participate in research, we may limit diversity in participation [5–8]. Shifting this culture is complex and somewhat uncharted territory, requiring careful assessment and evaluation. Our findings indicate that the program can result in modest but positive short-term outcomes. However, it is essential to note that mean scores on individual items presented in Figure 1 were relatively low, indicating that participants may need additional training. We are most encouraged by mid-term (9–12 months) follow-up findings that indicate behavior changes to promote inclusion. Despite the limitations of our small sample size, our findings that more than 76% of participants reported changes in their research practice due to their experience in the workshop (including increased action responding to bias and racism) are most meaningful and promising. Regarding the 24% of participants who did not report a change, we, unfortunately, know little about their experience as there were no open-ended responses from that group. We are, however, adding six-month follow-up interviews to our evaluation to explore this issue further.

Ultimately, no one intervention or educational program will achieve the goal of meaningful diversity in research participation. Indeed, as others have pointed out, there is a likely need for intervention at various levels and audiences [11,10]. *Just Research* is only a small part of this effort, but we are fortunate to be among others working to experiment with various approaches to researcher education [13–15]. Indeed, all these efforts have data demonstrating that change is possible, and perhaps more importantly, there is a willing audience for these interventions. We hope *Just Research* and our evidence base will contribute to ensuring that research is representative of patient populations and has the necessary diversity of participation to address health disparities.

### Limitations

As is the case with all studies, there are limitations to our work that are important to note. These include our relatively small sample size to date and reliance on self-report. In addition, our sample is dramatically skewed regarding gender (88.6%) identifying as women (88.6%). We will be working on understanding and correcting this imbalance as the program develops if it does not accurately represent the gender distribution of members of clinical research teams more broadly. It is important to note that the distribution in terms of race/ethnicity in our sample is not dissimilar to that of research teams. The majority of our participants identified as White (70%), which accurately reflects the characteristics of faculty and noninstructional academic staff at UW–Madison, which was 69.9% White in 2022 [22]. Interestingly, we saw an overrepresentation of participants identifying as Black or African American compared to the campus-wide faculty and nonacademic staff population (18.6% vs. 2.6%) [22]. The dramatic gender imbalance of the participants is less well understood. As workshop participants either self-select or are encouraged by other team members, it would seem that there is a striking gender imbalance either on research teams or in interest regarding inclusive research practices. This is a topic for future research which we are anxious to pursue. However, it is beyond the scope of this initial attempt to present *Just Research* outcomes and preliminary data.

## Supporting information

Passmore et al. supplementary material Passmore et al. supplementary material

## Supplementary material

The supplementary material for this article can be found at https://doi.org/10.1017/cts.2024.592

## References

[1] Varma T , Jones CP , Oladele C , Miller J. Diversity in clinical research: public health and social justice imperatives. J Med Ethics. 2023;49(3):200–203. doi: 10.1136/medethics-2021-108068. 35428737

[2] Boulware LE , Corbie G , Aguilar-Gaxiola S , et al. Combating structural inequities — diversity, equity, and inclusion in clinical and translational research. N Engl J Med. 2022;386(3):201–203. doi: 10.1056/NEJMp2112233. 35029847

[3] Knepper TC , McLeod HL. When will clinical trials finally reflect diversity? Nature. 2018;557(7704):157–159. doi: 10.1038/d41586-018-05049-5. 29743700

[4] Wilkins CH. Effective engagement requires trust and being trustworthy. Med Care. 2018;56(10 Suppl 1):S6–S8. doi: 10.1097/MLR.0000000000000953. 30015725 PMC6143205

[5] Manly JJ , Gilmore-Bykovskyi A , Deters KD. Inclusion of underrepresented groups in preclinical alzheimer disease trials-opportunities abound. JAMA Netw Open. 2021;4(7):e2114606. doi: 10.1001/jamanetworkopen.2021.14606. 34228130

[6] Gilmore-Bykovskyi A , Jackson JD , Wilkins CH. The urgency of justice in research: beyond COVID-19. Trends Mol Med. 2021;27(2):97–100. doi: 10.1016/j.molmed.2020.11.004. 33277159 PMC7855410

[7] Niranjan SJ , Martin MY , Fouad MN , et al. Bias and stereotyping among research and clinical professionals: perspectives on minority recruitment for oncology clinical trials. J Clin Oncol. 2019;37(27_suppl):152–152. 10.1002/cncr.32755 32147815

[8] Passmore SR , Kisicki A , Gilmore-Bykovskyi A , Green-Harris G , Edwards DF. There’s not much we can do…, researcher-level barriers to the inclusion of underrepresented participants in translational research. J Clin Transl Sci. 2022;6(1):e4. doi: 10.1017/cts.2021.876. 35154814 PMC8807123

[9] Passmore SR , Farrar Edwards D , Sorkness CA , Esmond S , Brasier AR. Training needs of investigators and research team members to improve inclusivity in clinical and translational research participation. J Clin Transl Sci. 2020;5(1):e57. doi: 10.1017/cts.2020.554. 33948278 PMC8057444

[10] Niranjan SJ , Durant RW , Wenzel JA , et al. Training needs of clinical and research professionals to optimize minority recruitment and retention in cancer clinical trials. J Cancer Educ. 2017;3(1):26–34. doi: 10.1007/s13187-017-1261-0. PMC5797508 28776305

[11] Boden-Albala B , Carman H , Southwick L , et al. Examining barriers and practices to recruitment and retention in stroke clinical trials. Stroke. 2015;46(8):2232–2237. doi: 10.1161/STROKEAHA.114.008564. 26185186 PMC5526461

[12] Boden-Albala B , Waddy SP , Appleton N , Kuczynski H , Nangle E , Parikh NS. Recruitment, inclusion, and diversity in clinical trials. In: The Science of Health Disparities Research. New Jersey, USA: John Wiley & Sons, 2021:413–428.

[13] Hartnett T. Minority research: building trust project aims to improve participation in strengthen capacity for investigators and IRBs. Res Pract. 2011;12(5):157.

[14] Kusnoor SV , Villalta-Gil V , Michaels M , et al. Design and implementation of a massive open online course on enhancing the recruitment of minorities in clinical trials – faster together. BMC Med Res Methodol. 2021;21(1):44. doi: 10.1186/s12874-021-01240-x. 33673809 PMC7936494

[15] Cranfill JR , Freel SA , Deeter CE , et al. Development and evaluation of a novel training program to build study staff skills in equitable and inclusive engagement, recruitment, and retention of clinical research participants. J Clin Transl Sci. 2022;6(1):e123. doi: 10.1017/cts.2022.456. 36259068 PMC9556271

[16] Passmore SR , Gerbitz A , Hancock GR , et al. “My blood, you know, my biology being out there…”: consent and participant control of biological samples. J Empir Res Hum Res Ethics. 2024;19(1-2):3–15. doi: 10.1177/15562646231222665. 38192107 PMC10957312

[17] Passmore SR , Fryer CS , Butler J , Garza MA , Thomas SB , Quinn SC. Building a “deep fund of good will”: reframing research engagement. J Health Care Poor Underserved. 2016;27(2):722–740. 27180705 10.1353/hpu.2016.0070 PMC5502676

[18] Thomas EV , Wells R , Baumann SD , et al. Comparing traditional versus retrospective pre-/Post-assessment in an interdisciplinary leadership training program. Matern Child Health J. 2019;23(2):191–200. doi: 10.1007/s10995-018-2615-x. 30173347

[19] Campbell DT , Stanley JC. Experimental and Quasi-Experimental Designs for Research. Boston: Houghton Mifflin Co., 1963.

[20] Howard GS , Ralph KM , Gulanick NA , Maxwell SE , Nance DW , Gerber SK. Internal invalidity in pretest-posttest self-report evaluations and a re-evaluation of retrospective pretests. Appl Psychol Meas. 1979;3(1):1–23. doi: 10.1177/014662167900300101.

[21] St. John BM , Hickey E , Kastern E , et al. Opening the door to university health research: recommendations for increasing accessibility for individuals with intellectual disability. Int J Equity Health. 2022;21(1):130. doi: 10.1186/s12939-022-01730-4. 36088334 PMC9464400

[22] Faculty & Staff. Accountability Dashboard. Published December 28, 2017. https://www.wisconsin.edu/accountability/faculty-and-staff/. Accessed December 5, 2023

