# Supplementary material for: Just Research: Evaluation findings of an educational program to promote inclusive research among investigators and research staff
Source: J Clin Transl Sci. 2024 Sep 16;8(1):e120. doi: 10.1017/cts.2024.592 (PMC11428049; doi:10.1017/cts.2024.592)
Supplement: Passmore et al. supplementary material [file S2059866124005922sup001.pdf]

Follow Up Survey - Just Research

Start of Block: Default Question Block

It's been a while since you participated in a Just Research Workshop. We're wondering how much this experience has stayed with you. Please, take a moment to let us know! Thank you!!

Please tell us how much you agree/disagree with the follow statements.

I've thought about the content of the Just Research Workshop several times since I participated in the workshop.

- ○ Strongly disagree (1)
- ○ Somewhat disagree (2)
- ○ Neither agree nor disagree (3)
- ○ Somewhat agree (4)
- ○ Strongly agree (5)

I've referred to my Just Research journal several times since I participated in the workshop.

- ○ Strongly disagree (1)
- ○ Somewhat disagree (2)
- ○ Neither agree nor disagree (3)
- ○ Somewhat agree (4)
- ○ Strongly agree (5)

I've talked to colleagues about the Just Research content or the workshop since I participated.

- ○ Strongly disagree (1)
- ○ Somewhat disagree (2)
- ○ Neither agree nor disagree (3)
- ○ Somewhat agree (4)
- ○ Strongly agree (5)

I've participated in other Just Research events (besides the workshop), such as seminars.

- ○ Strongly disagree (1)
- ○ Somewhat disagree (2)
- ○ Neither agree nor disagree (3)
- ○ Somewhat agree (4)
- ○ Strongly agree (5)

I've recommended the Just Research workshops or other events (such as seminars) to colleagues.

- ○ Strongly disagree (1)
- ○ Somewhat disagree (2)
- ○ Neither agree or disagree (3)
- ○ Somewhat agree (4)
- ○ Strongly agree (5)

I have been able to recognize different forms of implicit bias in my research practice.

- Strongly disagree (1)
- Somewhat disagree (2)
- Neither agree nor disagree (3)
- Somewhat agree (4)
- Strongly agree (5)

I have taken action when I perceived bias or racism on the research team or in interactions with participants.

- Strongly disagree (1)
- Somewhat disagree (2)
- Neither agree nor disagree (3)
- Somewhat agree (4)
- Strongly agree (5)

I think about community perspectives when considering a research project.

- Strongly disagree (1)
- Somewhat disagree (2)
- Neither agree nor disagree (3)
- Somewhat agree (4)
- Strongly agree (5)

I have taken steps to engage communities in research, including those who have been historically underrepresented.

- Strongly disagree (1)
- Somewhat disagree (2)
- Neither agree nor disagree (3)
- Somewhat agree (4)
- Strongly agree (5)

I have worked to incorporate more inclusive practices into my research.

- Strongly disagree (1)
- Somewhat disagree (2)
- Neither agree nor disagree (3)
- Somewhat agree (4)
- Strongly agree (5)

My experience with Just Research has led me to make changes in my work.

- ◯ Strongly disagree (1)
- ◯ Somewhat disagree (2)
- ◯ Neither agree nor disagree (3)
- ◯ Somewhat agree (4)
- ◯ Strongly agree (5)

Display This Question:

If My experience with Just Research has led me to make changes in my work. = Somewhat agree

Or My experience with Just Research has led me to make changes in my work. = Strongly agree

What specific changes have you made as a result of your participation in Just Research?

____

____

____

____

____

If you were to take a refresher course of Just Research now, what content do you think it should cover?

____

____

____

____

____

Is there anything else you think we should know about the Just Research program, now that you've had time to reflect?

____

____

____

____

____

End of Block: Default Question Block
